# Supplementary material for: Distinguishing artificial spin ice states using magnetoresistance effect for neuromorphic computing
Source: Nat Commun. 2023 May 4;14:2562. doi: 10.1038/s41467-023-38286-y (PMC10160026; doi:10.1038/s41467-023-38286-y)
Supplement: Supplementary file 1 — Supplementary Information [file 41467_2023_38286_MOESM1_ESM.pdf]

Distinguishing Artificial Spin Ice States using Magnetoresistance Effect for Neuromorphic Computing

Wenjie Hu[1,2,3], Zefeng Zhang[7,8], Yanghui Liao[1,2,3], Qiang Li[1,2,3], Yang Shi[1,2,3], Huanyu Zhang[1,2,3], Xumeng Zhang[7], Chang Niu[1,2,3], Yu Wu[1,2,3], Weichao Yu[1,5], Xiaodong Zhou[1,3,4,5], Hangwen Guo[1,3,4,5], Wenbin Wang[1,3,4,5], Jiang Xiao[1,2,3,4,5,6], Lifeng Yin[1,2,3,4,5,6,*] Qi Liu[7,*] and Jian Shen[1,2,3,4,5,6,*]

[1] State Key Laboratory of Surface Physics and Institute for Nanoelectronic Devices and Quantum Computing, Fudan University, Shanghai 200433, China

[2] Department of Physics, Fudan University, Shanghai 200433, China

[3] Shanghai Qi Zhi Institute, Shanghai 200232, China

[4] Shanghai Research Center for Quantum Sciences, Shanghai 201315, China

[5] Zhangjiang Fudan International Innovation Center, Fudan University, Shanghai 201210, China

[6] Collaborative Innovation Center of Advanced Microstructures, Nanjing 210093, China

[7] Frontier Institute of Chip and System, Fudan University, Shanghai 200438, China

[8] Research Institute of Intelligent Complex Systems and ISTBI, Fudan University, Shanghai 200433, China

*Emails: shenj5494@fudan.edu.cn, qi_liu@fudan.edu.cn, lifengyin@fudan.edu.cn

Contents :

1. The process of the demagnetization and MFM image.
2. COMSOL simulation for the tri-layer ASI spin structure.
3. The measured AMR and GMR effect of the Py base film and tri-layer structure.
4. The stability and reprogrammable character of the resistance value of eight spin states.
5. Magnetoresistance measurement of the tri-axial isolated islands ASI on Bi.
6. Transport characterization on the smaller nanomangets ASI tri-layer device.
7. ASI based reservoir computing device.

The process of the demagnetization and MFM image

The demagnetization protocols for tri-layer ASI device is to rotate the sample in an oscillating in-plane magnetic field.[1] The oscillating field stepped down is to decrease the remanent magnetic moment.

During demagnetization, our sample was at 120 rpm inside an oscillating magnetic field. The demagnetization procedure began at a field of 500 Oe which is well above the easy-axis coercive field of the nanomagnets.

After demagnetization, we can observe a chaotic distribution of eight spin states simultaneously in the MFM image. The yellow square I represents one of the two degenerate states of the Type A and Type F. The yellow square II represents one of the two degenerate states of the Type B and Type E. The yellow square III represents one of the two degenerate states of the Type C and Type H. And the yellow square IV represents one of the two degenerate states of the Type D and Type G.

The resistance of the sample after demagnetization is 133.302 Ω, which is slightly smaller than the average resistance of the eight spin configurations. And the relative value of the resistance in all states are shown below.

$$R_B < R_A < R_G < R_H < R_{DEMAGNETICZATION} < R_{AVERAGE} < R_D < R_C < R_F < R_E$$

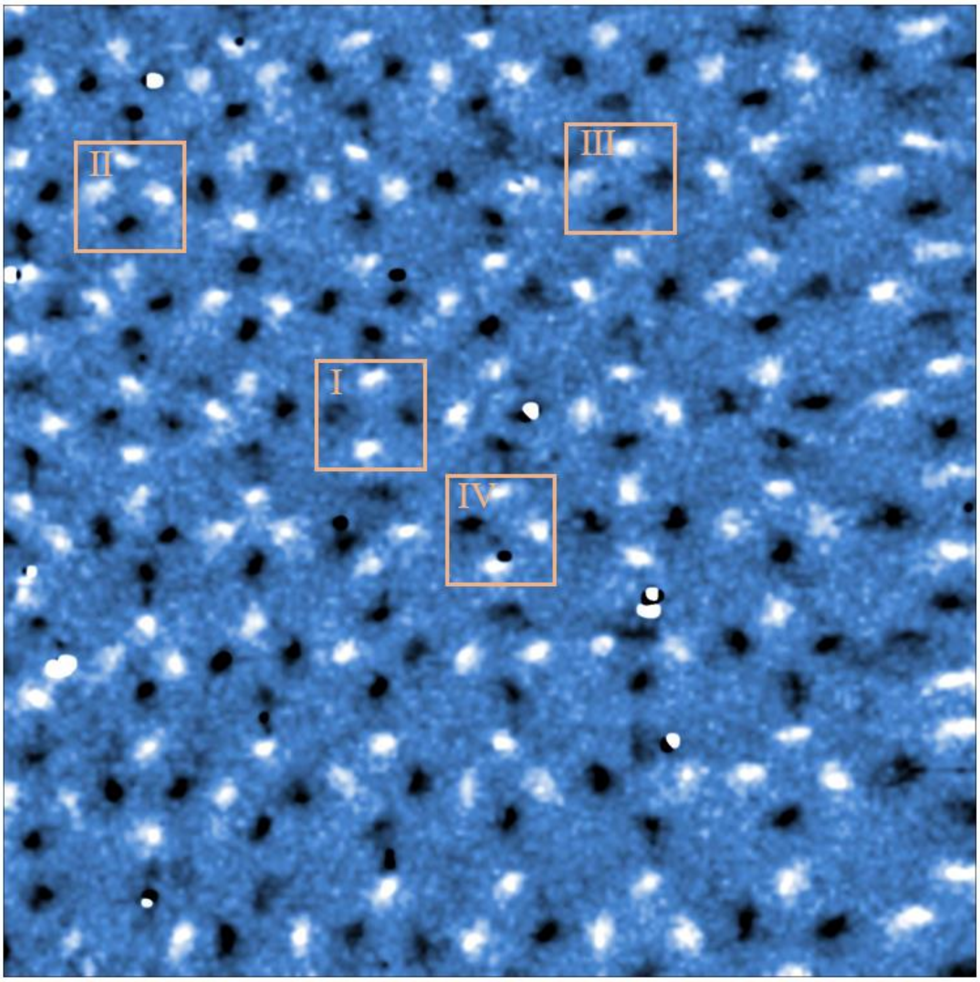

Supplementary Fig S1: MFM image of demagnetized state. It contains all the eight possible spin configurations.

COMSOL simulation

Based on the simulated spin structure of the tri-layer shown in Fig.3, we can calculate the resistivity behavior including both the AMR and GMR effects. According to the AMR effect, the local resistivity is

$$\rho_{tot} = \rho_{\perp} + (\rho_{||} - \rho_{\perp})\cos^2\alpha$$

where $\rho_{||}$ and $\rho_{\perp}$ are the resistivities for currents flowing parallel and perpendicular to the magnetization direction and α is the angle between the magnetization and the current. This formula can be expressed with the tensor

$$\rho_{[m]} = \rho_{\perp} I + (\rho_{||} - \rho_{\perp})\widehat{\mathbf{P}}_{[m]}$$

with I being the identity matrix and $\hat{P}_{[m]}$ being the projection operator $\widehat{\mathbf{P}}_{[m]} = \widehat{\mathbf{m}} \otimes \widehat{\mathbf{m}}$. Therefore, the conductivity tensor is derived as

$$\sigma_{[m]} = \frac{1}{\rho_{\perp}} I + (\frac{1}{\rho_{||}} - \frac{1}{\rho_{\perp}})\widehat{\mathbf{P}}_{[m]}$$

$$= \sigma_0 \left[I - \frac{6a}{6+a} \begin{pmatrix} m^2{}_x - \frac{1}{3} & m_x m_y & m_x m_z \\ m_y m_x & m^2{}_y - \frac{1}{3} & m_y m_z \\ m_z m_x & m_z m_y & m^2{}_z - \frac{1}{3} \end{pmatrix} \right]$$

where $\sigma_0 = \frac{1}{3\rho_{||}} + \frac{2}{3\rho_{\perp}}$ and the AMR ratio $a = \frac{2(\rho_{||} - \rho_{\perp})}{\rho_{||} + \rho_{\perp}}$ [2, 3].

Same method has been applied to the GMR effect. The resistivity of the GMR effect is

$$\rho_{tot} = \rho_P + (\rho_{AP} - \rho_P)\cos\varphi$$

where R_P (R_{AP}) is the resistivity when the magnetic direction of the upper layer and the base layer is parallel (antiparallel) and φ is the angle between the magnetization of the upper layer and the bottom layer. Thus the local conductivity can be approximate to

$$\sigma_{[\varphi]} \approx \frac{1}{\rho_{tot}} = \frac{1}{\rho_P + (\rho_{AP} - \rho_P)\cos\varphi}$$

As a two dimensional system, the region with the nanomagnets is dominated by the GMR effect and the region without the nanomangets is dominated by the AMR effect. The GMR effect we measured is larger than the AMR effect in this structure (see supplementary Fig.S2). And a 20 Oe in-plane field makes the magnetization of the Py base layer in good alinement, the AMR effect can be almost neglected in the calculated resistance.

The detail simulation micromagnetic spin configurations are shown below.

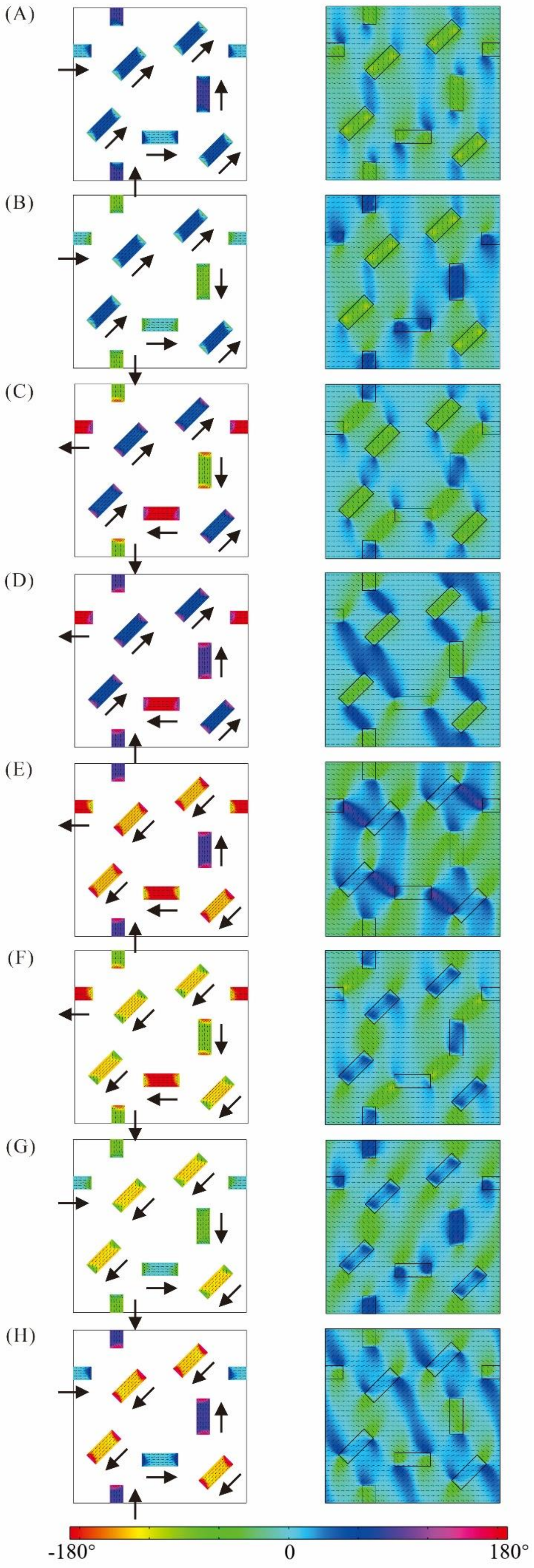
(A)
(B)
(C)
(D)
(E)
(F)
(G)
(H)
-180°
0
180°

Supplementary Fig S2: Detailed spin structure of the tri-layer ASI device simulated by micromagnetic modeling. (A) to (H) is corresponding to the type A to type H spin states in Fig. 1c. The left and right images represent simulated spin states of the ASI base unit and Py base layer. The color code represents the direction of the spins. $\theta = 0°$ is along the direction of the applied current.

The measured AMR and GMR effect of the Py base film and tri-layer structure

We measure the GMR and AMR value at room temperature. The GMR value is measured at the stack of Py (6 nm)/Cu (4 nm)/Py (16 nm). The bottom Py (6 nm)/Cu (4 nm) film is patterned into a 1000 μm x 1000 μm square. And the top Py (16 nm) film is patterned into a 470 μm x 170 μm wire. The in-plane magnetic field is swept along the current direction. The value of GMR is about 1.06%. And the AMR value is measured on a Py (10 nm) film. The AMR value is about 0.21%. All the transport behavior is measured with standard four-probe method. And the applied current is 20 μA.

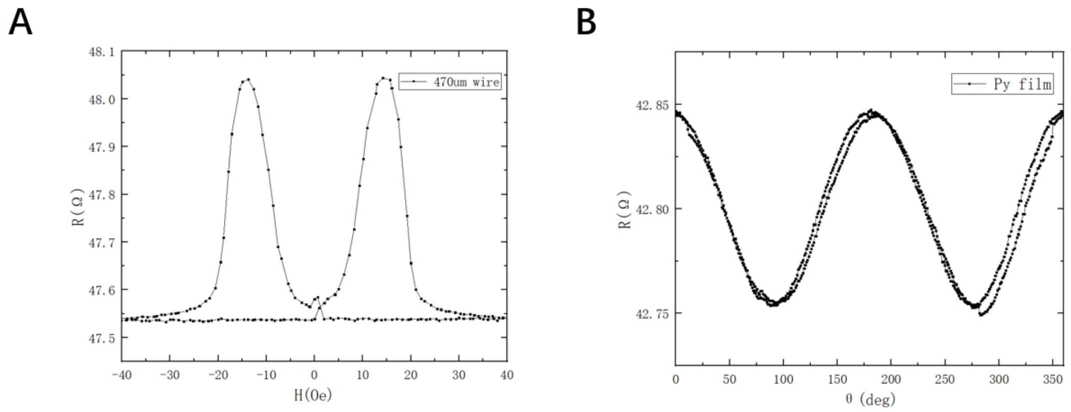

Supplementary Fig S3: The value of the GMR and AMR effect. (A) the GMR value of a wire with 470 μm (length) x 170 μm (width). (B) the AMR value of a 10 nm thick Py film.

The stability and reprogrammable character of the resistance value of eight spin states.

The ASI has a remarkable non-volatile character. The resistance of each long-range-ordered spin state shows a great stability at room temperature. The fluctuation of the resistance is smaller than 0.01395 Ω which can be neglected to the resolution of eight spin states. These non-volatile states can be held more than five hours with the standard deviation about 0.0029. Such stability makes it possible for the device to be used in practice.

The standard deviation of eight spin states resistance are listed in the Table 1.

Table 1: The standard deviation of eight spin state resistance.

Spin state	Type A	Type B	Type C	Type D	Type E	Type F	Type G	Type H
Standard deviation (Ω)	0.002341	0.002341	0.003416	0.002049	0.001757	0.001861	0.001898	0.00246

The largest standard deviation is about 0.0034, which represents a high consistency of the tri-layer GMR based ASI device. Besides, this structure also has outstanding repeatability. We note that spin state A can be reobtained from spin state type H by applying the magnetic field along θ=45°. For four cycle, the spin state type A presents an average resistance value with the standard deviation of 0.0012.

Table 2: The average resistance value of Type A for four cycle.

Cycle number	1	2	3	4
Average resistance for Type A (Ω)	133.2122	133.2107	133.2096	133.2120

We have examined the stability of the device by comparing the resistance distribution of the eight states with 4 H interval, which remains basically unchanged.

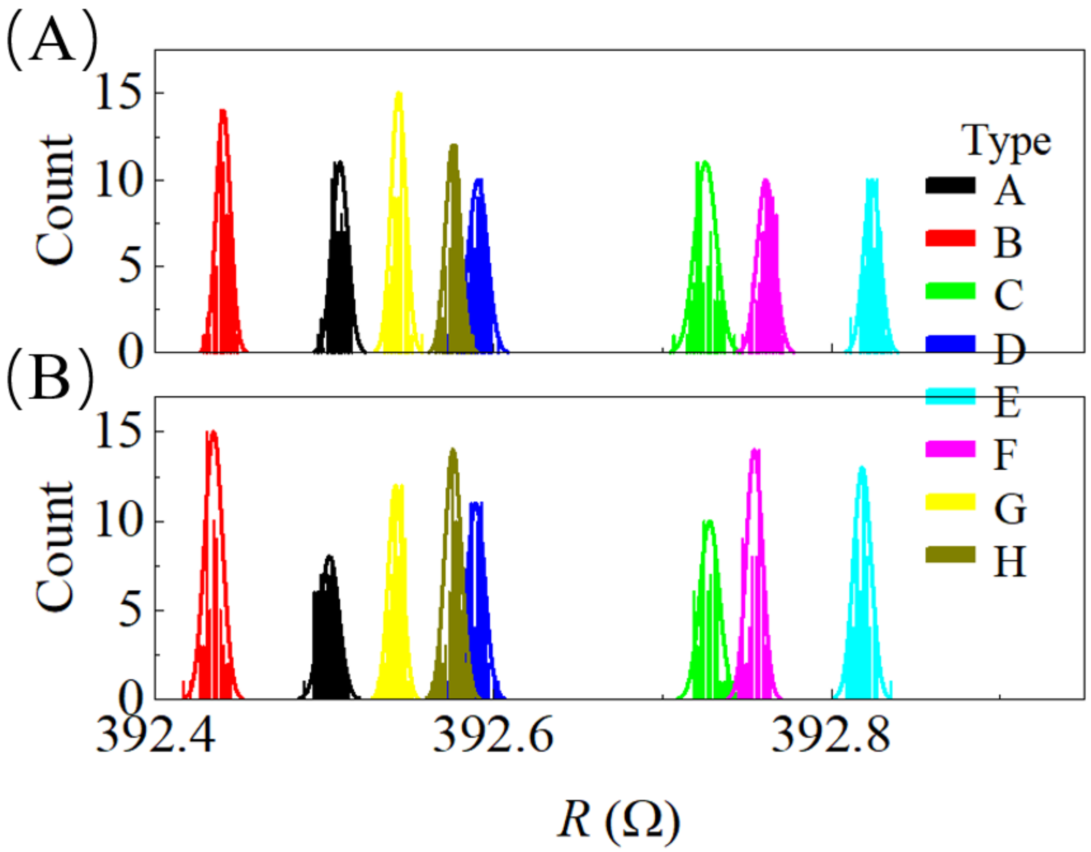

Supplementary Fig S4: the resistance distribution of the corresponding eight long-range ordered spin states with 4 hours interval.

Magnetoresistance measurement of the tri-axial isolated islands ASI on Bi.

We use the bilayer heterostructure containing the tri-axial ASI fabricated on top of 10 nm metal Bi film to test whether AMR effect can be used to distinguish eight spin states. Bi is less conductive than Py. So the current flow through the Py islands will represent their magnetization direction with the AMR effect. The permalloy islands have dimensions of 470 nm (l) × 170 nm (w) × 16 nm(d). The eight states are created by subsequent application of field (~200 Oe) along θ = 45°, 292°, 158°, 112°, 202°, 248°, 338°, and 112°, where θ represents the angle between the field direction and the horizontal direction as indicated in Fig.S4(a). and the magnetoresistance measurement shown in Fig.S4(b), gives no ability to distinguish between eight different spin states.

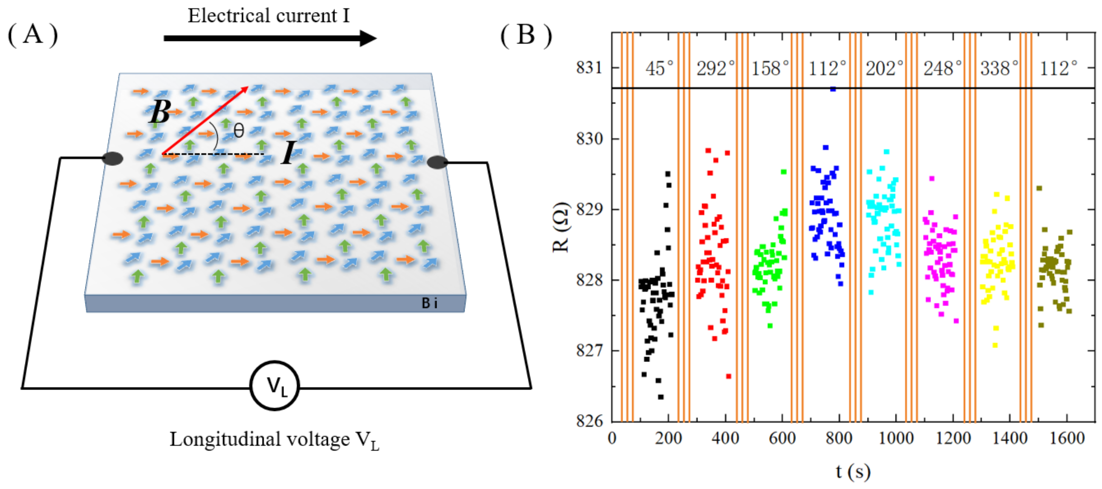

Supplementary Fig S5: (A) Schematic diagram of the tri-layer structure for transport measurement. The top tri-axial ASI layer is fabricated on the Bi base layer. The arrows in three colors represent the three axial sublattices. The black and red arrows indicate the directions of the applied current and the in-plane magnetic field. (B) Resistance of the eight long-range ordered spin configurations in the ASI bilayer structure with the sequence angle of in-plane magnetic field at room temperature.

Transport characterization on the smaller nanomangets ASI tri-layer device

We also design the device with different size of the nano-magnets in the tri-axial ASI array. The thickness of three layers is the same. The size of nano-magnets is 300 nm length and 100 nm width. And we get similar behavior of that with the size of 470 nm x 170 nm. This suggests that the behavior of longitudinal MR resistance is reflect directly to the corresponding spin configuration regardless of the specific size. The resistance distribution (full width at half maximum) is smaller than 0.0077 Ω, which is about the same range as that in the large size.

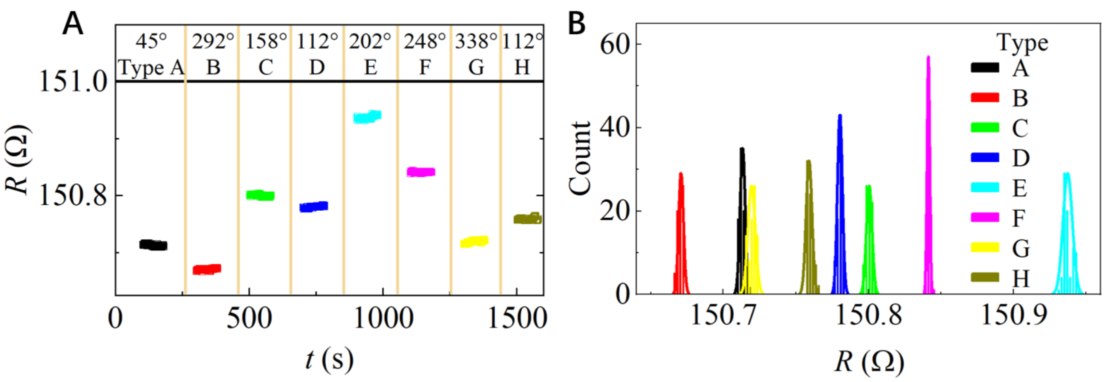

Supplementary Fig S6: (A) the corresponding longitudinal resistance of the 300 nm x 100 nm MR device. (B) The separated eight peaks of the histogram of the acquired resistance data.

ASI based reservoir computing device

As shown in Fig S6, we designed the ASI as the reservoir. The size of the ASI device is 200 um * 100 um. And each nanomagnet is 470 nm length and 170 nm width. We extended 8 electrodes on the device. The current is applied on the G7 and G8. The resistance elements in the matrix are selected from the G1 to G6.

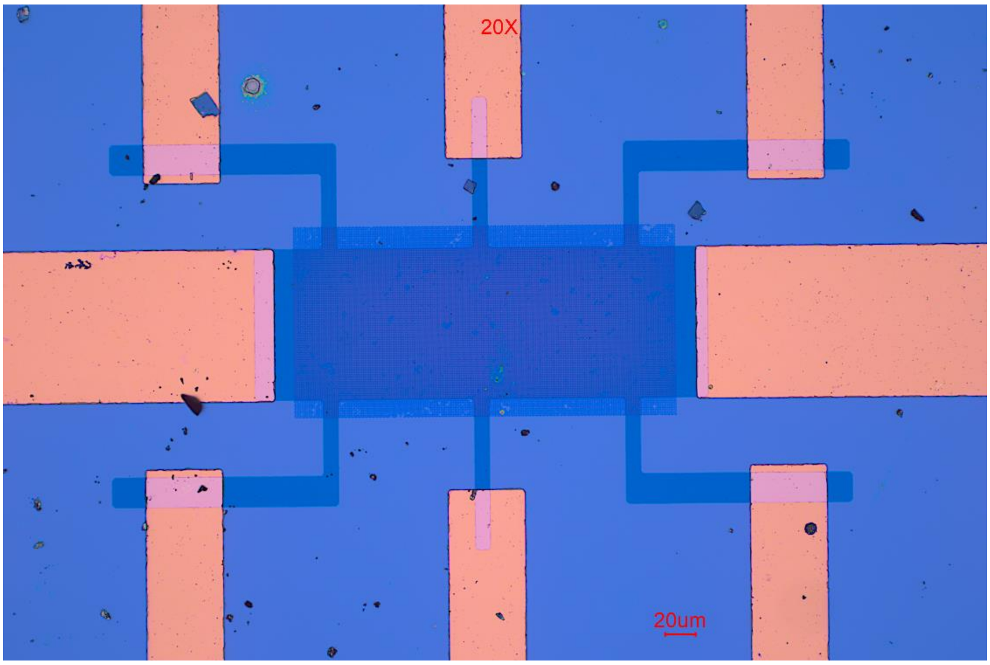

Supplementary Fig S7: Microscope image of the ASI based reservoir computing device.

The nonlinear behavior of the ASI device used for physical implementation of the RC system. We obtained the nonlinear curves of the input (angle of the in-plane magnetic field θ correspond to the current) and output signals (resistance) of 9 pairs of different electrodes. We map the input values linearly to the Angle of the magnetic field (0-360 degrees) in a plane. The resistance of the ASI reservoir to the nonlinear response of all input signals was measured in a reproducible chaotic initial state. The current is applied through the G7 and G8. The resistance elements selected from different electrodes (G1-G6) give the similar response but show the special difference in detail corresponding to the non-uniform spatial distribution.

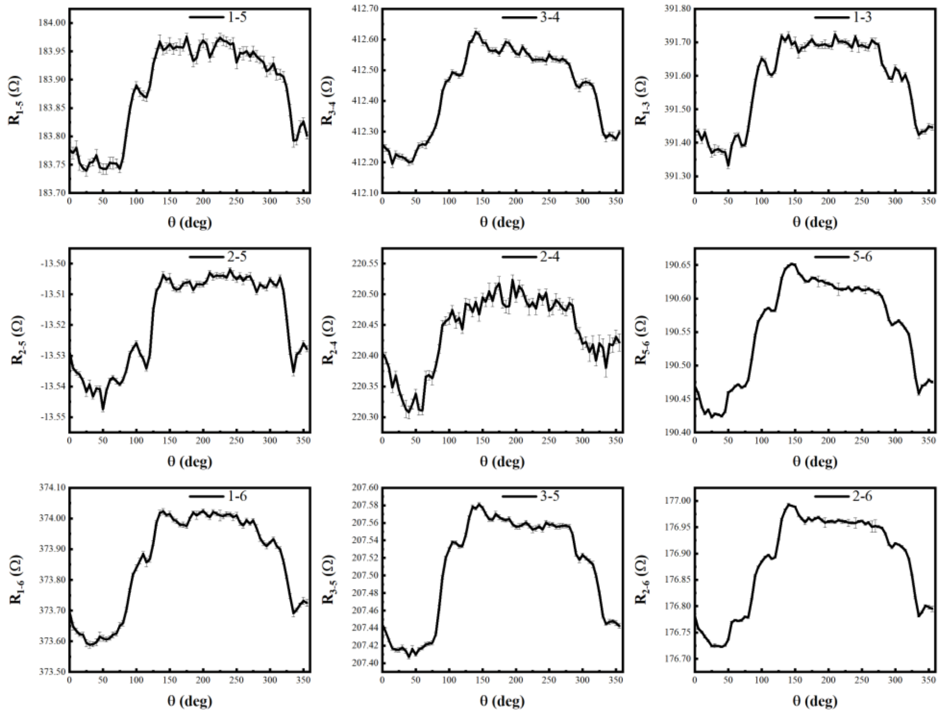

Supplementary Fig S8: the nonlinear curve of the output signal (resistance) for 9 pairs of different electrodes to the input signal (0-360deg).

The fading memory behavior of the ASI device used for physical implementation of the RC system.

We use the 8 long-range ordered spin configurations as the initial state to demonstrate fading memory behavior. N number of fields are subsequently applied to the ASI, where the field angle θ is randomly chosen from the eight angles used for preparing the eight long-range ordered spin states. While the resistance of the initial eight long-range ordered is clearly distinguishable (N = 1), with increasing N, resistance values, e.g. R1-3, shows a clear tendency towards convergence to the same resistance value. This shows that the recent stimulus has a greater effect on the final spin configuration than the remote history.

Under a modest field, the physical origin of the fading memory behavior of the present ASI system is the randomization of spin configuration after a subsequently applied random fields. Although the eight long-range ordered states are distinctly different, their spin configurations will be more and more randomized with increasing number of random fields applied leading to similar resistance value. Under a large field, the fading memory behavior should be caused by the tendency towards one single spin state.

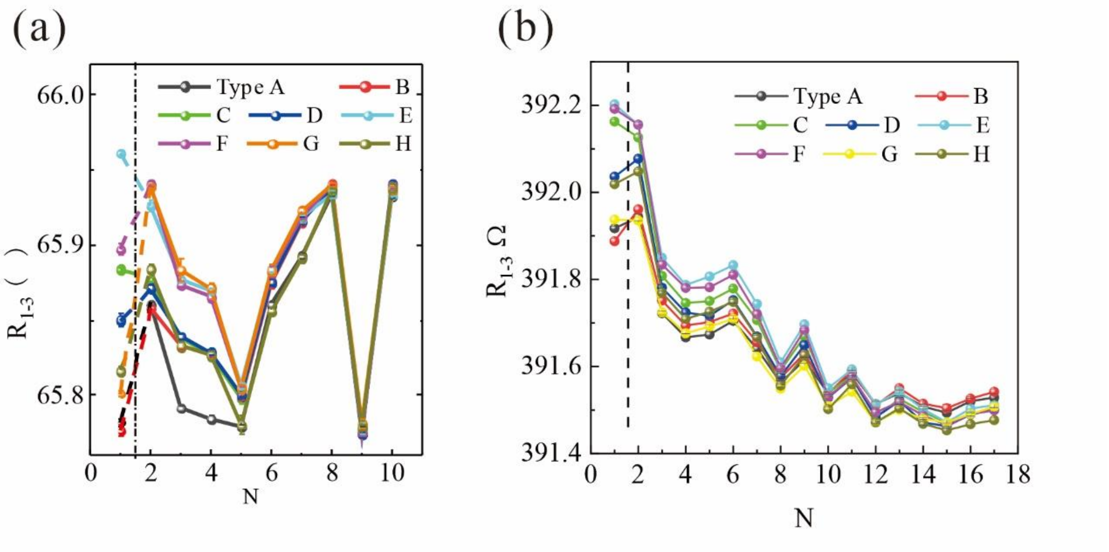

Supplementary Fig S8: ASI based reservoir computing device has fading memory. (a) under a relative large field ~ 70 Oe (b) under a relative small field ~ 50 Oe

The long-term memory behavior of the ASI device used for physical implementation of the RC system.

Random stimulus makes the reservoir have a large number of spin states. Except from the fading memory mentioned in the text, ASI reservoir has great long-term memory. Benefit from the intrinsic strong coupling interaction and non-volatile character, ASI reservoir shows a great history dependence behavior. We select the 10 group of resistance data before and after the same magnetic stimulus at θ = 292° in a long series of random selected excitations. Each stimulus applied an in-plane magnetic sweep field of 70 Oe at 292°. And the resistance is measured after the sweep field at zero field at θ = 0°. Before applying the stimulus, the ASI has different resistance values representing the device in a different spin states with different history. When the same excitation is applied, the reservoir obtains different resistance values. This is an important finding that ASI reservoir exhibits different responses to same stimuli based on history. It is important to emphasize that fading memory shows the process of forming a similar state after a certain amount of the same excitation from different initial states. While the long-term memory is that the reservoir with different history in different states will show different responses when the same stimulus is applied to them.

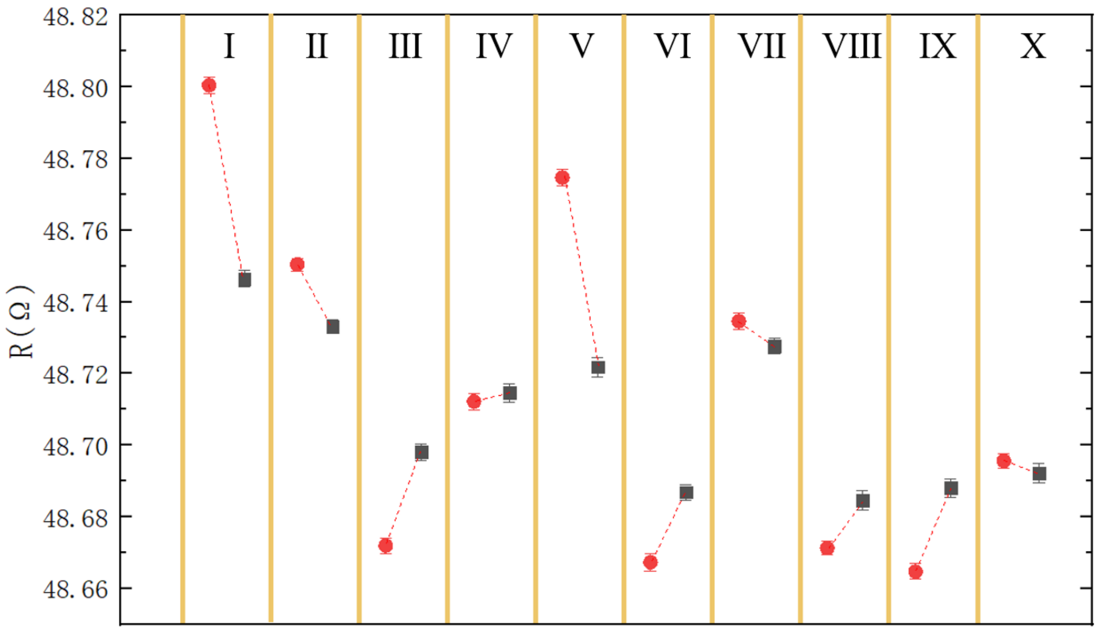

Supplementary Fig S10: Long-term memory behavior in ASI reservoir. 10 group selected data is shown in the image. The red dot is the resistance state before the magnetic stimulus at θ = 292°. And the black dot is the resistance state after the identical magnetic stimulus. The reservoir gives different responses to the same stimuli based on history.

ASI-based single node RC configuration

In this work, the RC we build involves the common masking/time-multiplexing process, the sampling of state points in time domain and the conversion of these state points into virtual nodes of the RC of each frame in single node RCs.[4] The novelty and difference from other forms of single node RCs here is that we also utilize the electrode terminals on the single ASI device and their different nonlinear response to the same external input, making use of both temporal states as well as spatial nodes in the ASI-based RC.

Time-multiplexing/Masking Process: Assume that the magnetic field loop operation time is θ, and that the temporal state of the resistance between a pair of electrodes (a physical node) is immediately read out after the completion of each operation. The input is preprocessed before the time-multiplexing process. For continuous input, we adopt a 'sample and hold' process to obtain a step input, while for discrete input, we just hold each data point for a period of time.[4] We will refer to the preprocessed input as the original input before time-multiplexing process below. The time-multiplexing/masking is to process the original input to get a train of signal with higher frequency of change as the actual input into the RC system. Consider that the actual input (of only one dimension) into the ASI device changes N_v times when the original data input (of L dimensions) is kept same, keeping the time interval between different original data input to be $\tau = N_v\theta$. We can write the relationship in the following form:

$$J(t) = \boldsymbol{I}(t) \times \boldsymbol{M}(t)$$

where $\boldsymbol{I}(t)$ (time-variant row vector in the form of $1 \times L$) is the original input data with time length τ of each step, $\boldsymbol{M}(t)$ (time-variant column vector in the form of $L \times 1$) in the form is the time-variant mask vector as a step-like function of t with time length θ of the smallest step and a period of τ. And the $J(t)$ obtained is the masked input to RC, also a step-like function of time t with the smallest step θ. In the numerical simulation of the single node RC, we actually adopt the discrete form of $J(t)$, with sampling rate of $\frac{1}{\theta}$, as the actual input to the RC considering the iterative nature of the RC we build. We later denote the actual input as $J_{p,q}$, referring to the $(p \cdot N_v + q)$th data point in the train of discretized $I(t)$, with $0 < q \; < \; N_v$; $N_v, p, q \in \mathbb{N}$.

Reservoir Computing: We consider to transform each $J_{p,q}$ into corresponding operation to the ASI device. Here a standard operation to the device is one round of triangular sweeping of the magnetic field in a given direction. Here we choose to fix the maximal strength of the magnetic field during sweeping and change the direction of the magnetic field in response to the changing actual input. In order not to introduce other forms of nonlinearity to the whole RC system, we map the original input linearly to the range of all possible directions, denoted as an angle ranging from $[0, 2\pi]$. Considering that the ASI device would possibly operate in a rather chaotic state during the computation process of the RC (since there are only eight long-range ordered state in this ASI device), we utilize the experimentally measured response of the change in the resistance between ASI device's different pairs of electrodes from a chaotic initial state of the device. Here we use the linear interpolation to fill the whole nonlinear response of the resistance of each pair of electrodes. Thus, based on the results from experiments, we modify a previously proposed physical RC model[5] and model the iteration process of RC behind the operations on the ASI device as follows:

$$R_{i,t+1} = R_{i,t} + k \cdot f_i(H) \cdot (R_{i,target}(\beta_{t+1}) - R_{i,t}) \qquad \text{(S1)}$$

where $R_{i,t+1}$ is the resistance between ith pair of electrodes (called ith node for simplicity in the following) of the next step, $R_{i,t}$ is the current resistance of ith node, $R_{i,target}$ describes the nonlinear response of the ith node to different angle β_{t+1} of the magnetic field, f_i is the response corresponding to the strength of the magnetic field, H is the maximal strength of the applied magnetic field, k is a constant. Here we require that $f_i(H) = 0$ when $H = 0$. Here, the range of $R_{i,t+1}$, $R_{i,t}$ and $R_{i,target}$ are all normalized to the range $[-1,1]$ to obtain only the trend in the change of R. Since we only consider the input mapped to the direction of the magnetic field, therefore we also consider all $f_i(H)$ to be the same and constant. We give a specific form of $f_i(H) = \frac{\alpha|H|}{\alpha|H|+1}$ in our numerical simulations. The model we propose here provide possible answers to the seemingly conflict between nonvolatility and short-term memory, and succeed in reproducing the experiment results characterizing the device's essential properties for being utilized as RC in numerical simulations, and performs well in several RC tasks. Here in the training of the RC in specific tasks, we adopt ridge regression to obtain the resulting read-out weight matrix (output layer). The specific setting of the number N_p of physical node used, the number N_v of virtual nodes sampled from each physical node and the ridge parameter λ_r may differs across different tasks.

Modelling the Results from Experimental Measurement: Traditionally, RC requires a dynamical system with echo state properties, mainly the fading memory and the nonlinear mapping into space of higher dimension.[4,6] For physical devices, the fading memory refers to a decay property in most cases.[5,7-9] However, in the case of RC based on ASI devices, it is not the case. In the RC built with ASVI device, we have seen the usage of a type of nonvolatile device in successful RC application. The authors claim the converging behavior starting from different initial states under the same train of external input to be the essential fading memory property for RC. Here we use the model described in S1 to model the similar behavior we observed in the ASI device proposed in this paper and explain the effectiveness of the 'fading memory of nonvolatile device' in realizing RC. First, in modelling the converging behavior, we assign the same train of input to the model in S1, but starting from different initial state points, obtaining similar converging behavior as shown in Fig S11. It is noteworthy that the model S1 also describes well the nonvolatile nature of the device. When assuming the external field strength H to be 0, it is clear that $R_{i,t} = R_{i,0}$ since $f_i(H) = 0$. When there is external input, the model S1 can be written in the following form:

$$R_{i,t+1} = (1 - k \cdot f_i(H)) \cdot R_{i,t} + k \cdot f_i(H) \cdot R_{i,target}(\beta_{t+1}) \qquad \text{(S2)}$$

In the specific form $f_i(H) = \frac{\alpha|H|}{\alpha|H|+1}$, we can write an equivalent decay parameter:

$$r_{decay} = 1 - k \cdot \frac{\alpha|H|}{\alpha|H| + 1}$$

By choosing proper parameters k, α, when $0 < r_{decay} < 1$, the model appears decaying, meeting the requirement for short-term memory. Since we consider H to be constant, S2 could be written in the following simpler form:

$$R_{i,t+1} = r \cdot R_{i,t} + I_{t+1} \qquad \text{(S3)}$$

Now we revisit the converging behavior, consider two different initial states $R_{i,0}, \tilde{R}_{i,0}$, first we consider the states at $t = 1$, the difference of $R_{i,1}$ and $\tilde{R}_{i,1}$:

$$\tilde{R}_{i,1} - R_{i,1} = r \cdot (\tilde{R}_{i,0} - R_{i,0})$$

We consider $0 < r < 1$, thus obtaining declining state difference with iteration going on. After n times of iteration under the same train of input $[I_1, I_2, I_3, \dots, I_n]$, the difference between the two resulting states:

$$\tilde{R}_{i,n} - R_{i,n} = r^n \cdot (\tilde{R}_{i,0} - R_{i,0})$$

And there is limit $\lim_{n \to +\infty} r^n = 0$, thus after enough rounds of iteration, the states of the RC would converge to a state defined by inputs from the recent history, regardless of its initial states and inputs far in the past, exhibiting the fading memory property with the nonvolatile device.

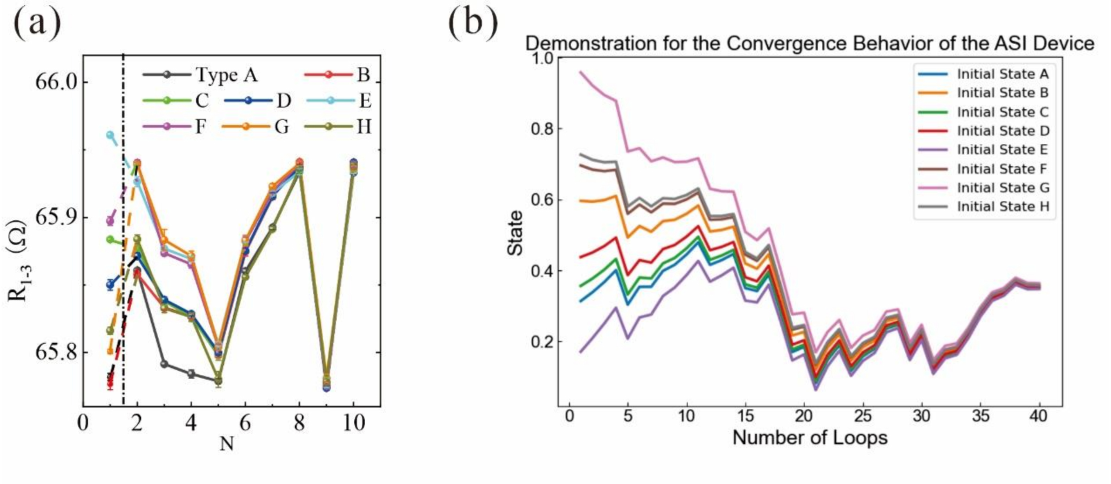

Supplementary Fig S11: Fading memory obtained from experiments and models on ASI based reservoir computing device. (a) experiment (b) model

MG One-step Prediction

In the Mackey-Glass one-step prediction task,[10] we generate the original input series with the following system:

$$\frac{dx(t)}{dt} = -b \cdot x(t) + \frac{ax(t-\tau)}{1 + (x(t-\tau))^c}$$

where $a = 0.2, b = 0.1, c = 10, \tau = 18, dt = 1$ with initial setting $x(t) = 0.01$ for $t \in [-\tau, 0]$. We generate total length of 4000, and split the training/testing in 0.5/0.5. The elements of the mask are binary, either -1 or 1. In the RC settings, we set $N_v = 4$, $N_p = 9$ to generate a frame of RC states with state vector of 36 dimensions. In the ridge regression process, we set $\lambda_r = 10^{-8}$.

Sun-spot One-step Prediction

The sun-spot data series is obtained from the WDC-SILSO daily sun-spot data, from 1818.1.1 to 2022.5.31.[11] We take the slice from 66000th to 74000th records of the daily data, creating a data series of 8000 original data points, still with training/testing split in 0.5/0.5. The elements of the mask are binary, either -1 or 1. In the RC settings, we also set $N_v = 4$, $N_p = 9$, with smaller $\lambda_r = 10^{-10}$.

Reference:

1 Wang, RF and, et al. Demagnetization protocols for frustrated interacting nanomagnet arrays. Journal of applied physics 101, 09J104 (2007).

2 Krüger, Benjamin. Current-driven magnetization dynamics: analytical modeling and numerical simulation. Diss. Staats-und Universitätsbibliothek Hamburg Carl von Ossietzky, (2011).

3 Prychynenko, Diana, et al. Magnetic skyrmion as a nonlinear resistive element: a potential building block for reservoir computing. Phys. Rev. A 9, 014034 (2018).

4 Appeltant, Lennert, et al. Information processing using a single dynamical node as complex system. Nature communications 2, 1-6 (2011).

5 Zhong, Yanan, et al. Dynamic memristor-based reservoir computing for high-efficiency temporal signal processing. Nature communications 12, 1-9 (2021).

6 Jaeger, Herbert. Adaptive nonlinear system identification with echo state networks. Advances in neural information processing systems 15 (2002).

7 Gartside, Jack C., et al. Reconfigurable training and reservoir computing in an artificial spin-vortex ice via spin-wave fingerprinting. Nature Nanotechnology 17, 460-469 (2022).

8 Du, Chao, et al. Reservoir computing using dynamic memristors for temporal information processing. Nature communications 8, 1-10 (2017).

9 Milano, Gianluca, et al. In materia reservoir computing with a fully memristive architecture based on self-organizing nanowire networks. Nature Materials 21, 195-202 (2022).

10 Moon, John, et al. Temporal data classification and forecasting using a memristor-based reservoir computing system. Nature Electronics 2, 480-487 (2019).

11 https://www.sidc.be/silso/datafiles-old, International Sunspot Number Monthly Bulletin and online catalogue, 1818-2022
